# Mechanical Activation of the β_2_-Adrenergic Receptor by Meningococcus: A Historical and Future Perspective Analysis of How a Bacterial Probe Can Reveal Signalling Pathways in Endothelial Cells, and a Unique Mode of Receptor Activation Involving Its N-Terminal Glycan Chains

**DOI:** 10.3389/fendo.2022.883568

**Published:** 2022-05-02

**Authors:** Stefano Marullo, Mark G. H. Scott, Hervé Enslen, Mathieu Coureuil

**Affiliations:** ^1^ Université de Paris, Institut Cochin, INSERM U1016, CNRS UMR 8104, Paris, France; ^2^ Université de Paris, Institut-Necker-Enfants-Malades, INSERM U1151, CNRS UMR 8253, Paris, France

**Keywords:** meningococcus, β_2_-adrenergic receptor, β-arrestin, N-glycans, sialic acid, mechano-transduction, pilin, meningitis

## Abstract

More than 12 years have passed since the seminal observation that meningococcus, a pathogen causing epidemic meningitis in humans, occasionally associated with infectious vasculitis and septic shock, can promote the translocation of β-arrestins to the cell surface beneath bacterial colonies. The cellular receptor used by the pathogen to induce signalling in host cells and allowing it to open endothelial cell junctions and reach meninges was unknown. The involvement of β-arrestins, which are scaffolding proteins regulating G protein coupled receptor signalling and function, incited us to specifically investigate this class of receptors. In this perspective article we will summarize the events leading to the discovery that the β_2_-adrenergic receptor is the receptor that initiates the signalling cascades induced by meningococcus in host cells. This receptor, however, cannot mediate cell infection on its own. It needs to be pre-associated with an “early” adhesion receptor, CD147, within a hetero-oligomeric complex, stabilized by the cytoskeletal protein α-actinin 4. It then required several years to understand how the pathogen actually activates the signalling receptor. Once bound to the N-terminal glycans of the β_2_-adrenergic receptor, meningococcus provides a mechanical stimulation that induces the biased activation of β-arrestin-mediated signalling pathways. This activating mechanical stimulus can be reproduced in the absence of any pathogen by applying equivalent forces on receptor glycans. Mechanical activation of the β_2_-adrenergic receptor might have a physiological role in signalling events promoted in the context of cell-to-cell interaction.

## Introduction

### A Signalling Pathogen With Missing Receptors

Among the multiple pathogens causing infectious diseases in humans, some are more daunting than others. *Neisseria meningitidis (Nm*, also known as meningococcus) belongs to this category. Indeed, this Gram-negative extracellular bacterium can kill a perfectly healthy person in a few hours. Meningococcus behaves in most cases as a commensal non-pathogenic bacterium of the human nasopharynx (1). However, when it leaves its “ecological niche” to penetrate into the blood stream it can infect the meninges, the membranes which envelop the brain and spinal cord, causing cerebrospinal meningitis (2). Moreover, in cases of invasive disease often associated with high bacteraemia (3), meningococcus disseminates into tissues promoting inflammation and coagulation activation, leading to extensive necrotic *purpura* and sepsis (4).

A key event in meningococcus tissue dissemination is its capacity to interact with host endothelial cells, allowing the rapid colonization of microvessels. The interaction with endothelia largely relies on bacterial filamentous appendages known as type-4 pili (TFP) (5, 6). The chemical inhibition of TFP using drugs that promote their disassembly markedly reduces meningococcus pathogenicity (7). TFP are made of polymers of protein subunits called pilins, which assemble in long helical structures. PilE, the most abundant pilin within the polymers, can assemble with minor (less abundant) PilV, PilX and ComP pilins (8). The PilC protein, presumably located at the tip of TFP is involved in pili adhesiveness along with PilV (9, 10). Pili are submitted to cycles of extension and retraction that are controlled by 2 ATPases: the PilF ATPase regulates pilin assembly, whereas the PilT ATPase is responsible for TFP disassembly and retraction. The dynamics of TFP elongation and retraction, allows the bacterium to crawl at the surface of endothelial cells and to adapt to microvessel geometry (11). Recently, TFP retraction was shown to promote the release of TFP-dependent contacts between bacteria facilitating sustained bacteremia (12). Although not directly measured for *Nm*, it has been established in a study on the close pathogen *N. gonorrhoeae* that TFP retraction can generate proportionally high forces in the nanonewton range (13).

The interaction of TFP with both epithelial and endothelial cells promotes the activation of multiple signalling cascades, the visible hallmark of this phenomenon being the bulk accumulation under bacterial colonies of membrane-associated proteins, which form a honeycomb shaped “cortical plaque” (14). However, signalling pathways Induced by TFP in endothelial and epithelial cells are different, likely involving different receptors (15).

In endothelial cells, meningococcus induces the accumulation of adhesion molecules, junctional proteins, cytosolic signalling proteins and cytoskeletal proteins, such as ezrin and actin (16), with two principal pathophysiological consequences. The first is the creation of actin-containing microvilli-like structures perpendicular to the endothelial surface, which protrude between the bacteria of a growing colony and stabilize it under the blood flow (17). The second is the formation of ectopic intercellular junctional domains at the site of bacteria-host cell interaction and a subsequent depletion of junctional proteins at the cell-cell interface, with opening of the intercellular junctions of the brain-endothelial interface (18). Early studies identified several signalling mechanism upstream of these cellular changes, including: local activation of Cdc42 and Rho GTPases (16); Src activation (19) and subsequent Src-dependent phosphorylation of cortactin, an actin-binding protein that controls actin polymerization; activation of the phosphoinositide-3-kinase (PI3K)/Rac1 pathway (20). However, the host cell receptor(s) activated by the pathogen upstream of these signalling events remained elusive for many years.

## An Endothelial-Cell Receptor Couple Working Together: One for Signalling, One for Early Adhesion

The accumulation in the cortical plaque of different protein types was consistent with the involvement of one or more scaffolding protein(s) that would be recruited by a putative meningococcus-activated receptor. Two such scaffolding proteins, β-arrestin 1 and 2 (βarr1 and βarr2), which are principally involved in G protein coupled receptor (GPCR) regulation and signalling, appeared as plausible candidates. Previous studies had shown that βarrs were essential for the co-localization of stimulated GPCRs, the actin-binding protein filamin-A and active ERK in membrane ruffles (21). Moreover, βarrs were shown to participate in the activation of CDC42 (22), PI3K (23), and Src (24).

The hypothesis of a role for βarrs in *Nm*-induced signalling in host endothelial cells was validated. In monolayers of hCMEC/D3 cells, a human brain endothelial cell line, which stably maintains phenotypic features of BBB in culture (25, 26), capsulated (pathogenic) piliated meningococci induced the accumulation in cortical plaques of both exogenous βarr2-GFP and endogenous βarr2, together with the proteins that were commonly found under bacterial colonies (27). Inhibition of βarrs with specific siRNAs inhibited the formation of cortical plaques (visualized by ezrin staining), demonstrating that βarr recruitment to the putative unknown *Nm* receptor was actually an upstream signalling event. Interestingly, siRNA-mediated inhibition of GRK2, a kinase which specifically phosphorylates GPCRs thus providing docking sites for βarrs, prevented βarr translocation under bacterial colonies and cortical plaque formation. This finding represented a strong experimental argument for the involvement of a GPCR in meningococcus signalling. Multiple studies combining loss and gain of function assays demonstrated that the missing signalling receptor in hCMEC/D3 cells was the β_2_-adrenergic receptor (β_2_AR) and provided a comprehensive scenario of the signalling events elicited by *Nm* following its adhesion to human endothelial cells (27). After binding onto endothelial cells, *Nm* TFP interact with the host cell β_2_AR, leading to its βarr-biased activation, independently of the activation of cognate heterotrimeric Gα_s_ protein and of its downstream adenylyl cyclase-cAMP pathway. The pilus components PilE and PilV were identified as the specific “bacterial ligands” involved in β_2_AR activation. Once translocated to β_2_ARs, βarrs drive the 2 major events involved in *Nm* tissue infection: docking and activation of Src, leading to the formation of actin-rich cellular protrusions, which stabilizes bacterial colonies under the blood flow; accumulation under bacterial colonies of proteins, such as VE-cadherin and p120-catenin that are normally located in intracellular junctions, which, once depleted, become permeable to bacteria. However, from the studies above it also emerged that the β_2_AR was not competent for the initial adhesion of the pathogen, corroborated by the fact that β_2_AR-depleted cells still support initial pilus-mediated *Nm* adhesion. These observations were confirming previous reports indicating that TFP-mediated adhesion and signalling are two independent events (14).

**Figure 1:**
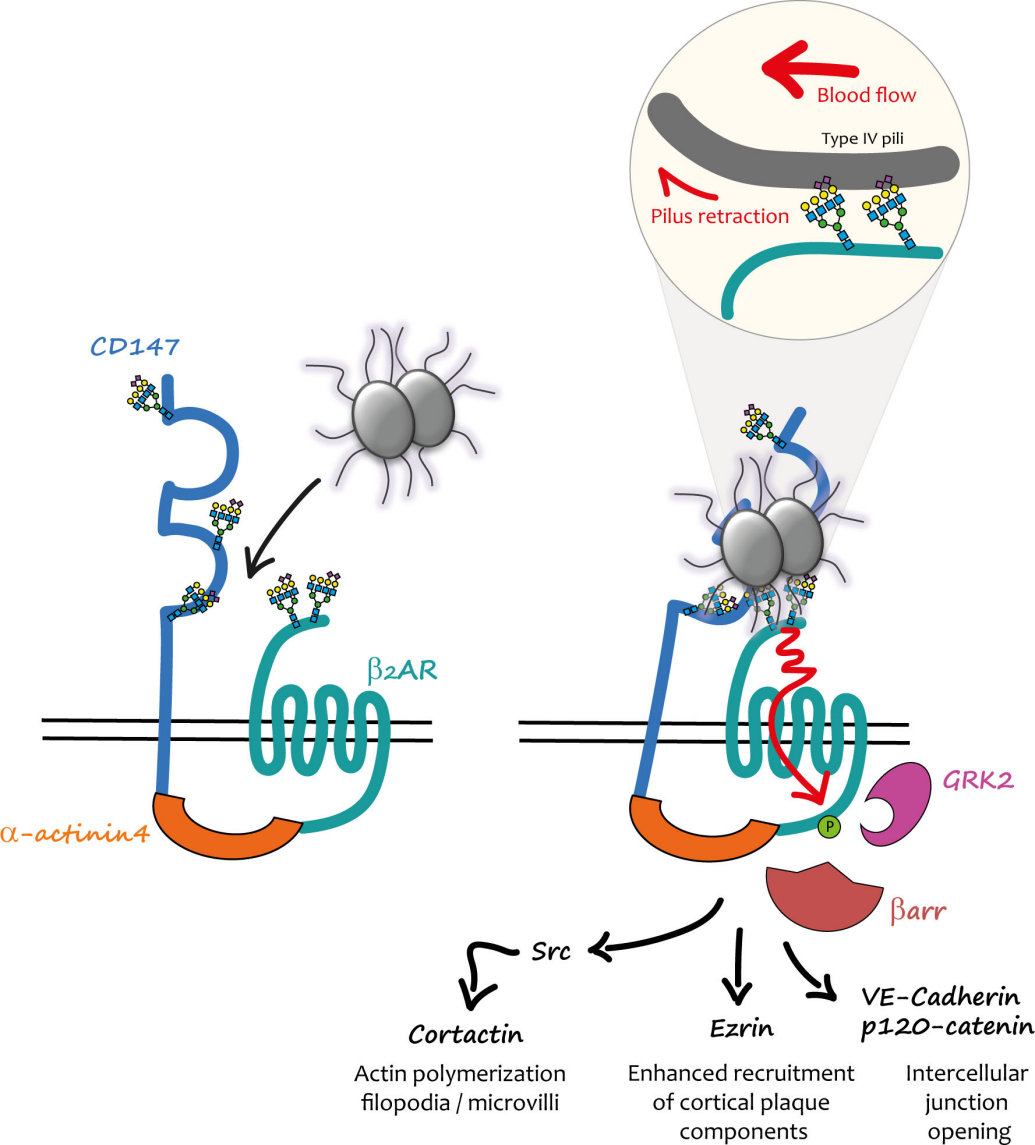
Mechanical activation of the CD147-β_2_AR heterodimer by meningococcus in endothelial cells. (Left) In endothelial cells CD147 and β_2_AR are pre-assembled into heterodimers stabilized by α-actinin4; the physiological role of these complexes is still unknown. When meningococci penetrate into the blood stream, they interact with these heterodimers *via* the PilE and PilV pilins of their TFP. (Right) The first interaction presumably involves CD147 and is accountable for its “early adhesion”. Terminal Neu5Ac (sialic acid) of the N-glycan chains present in the proximal IgG domain of CD147 constitutes the binding site of TFP pilins. Other TFP interact *via* PilE and PilV with the terminal Neu5Ac of close β_2_AR N-glycan chains. TFP retraction powered by PilT and forces generated by the blood flow concur to the mechanical activation of the β_2_AR (enlarged inset). The conformational change induced by this activation produces a cascade of signalling events promoted by βarr translocation to GRK2-phosphorylated β_2_AR. Once the “stable adhesion” of bacterial colonies is achieved meningococci cross the endothelium through intercellular spaces.

A differential, quantitative, large-scale analysis of gene expression was conducted in a cell line, which only become permissive for the attachment of piliated capsulated meningococci after phorbol ester 12-O-Tetradecanoylphorbol-13-acetate treatment. This strategy led to the identification of the immunoglobulin superfamily member CD147 (also known as basigin or emmprin) as a brain microvasculature early adhesion receptor for meningococcus (28). PilE and PilV pilins specifically mediate the interaction of the pathogen with the C-terminal part of the CD147 Ig domain, showing that the same bacterial ligands are involved in the interaction with the signalling and the adhesion receptor. These findings indicated that CD147 and β_2_AR might cooperate to promote firm *Nm* adhesion and subsequent activation of signalling events, and suggested a potential functional connection between these receptors that might pre-exist pathogen infection (**Figure 1**). 

The hypothesis of a spatio-temporal coordination between adhesion to CD147 and β_2_AR stimulation by *Nm* TFP was specifically addressed in a subsequent study (29). CD147 and β2AR were found in pre-existing complexes at the cell surface of endothelial cells independently of bacterial infection. This interaction was stabilized by the cytoskeletal protein actinin-4, which in addition promoted the assembly of highly ordered clusters of receptor complexes in response to meningococcus adhesion. *In vivo*, under blood flow, this multi-molecular assembly process likely provides sufficient binding strength for the initial interaction of TFP with CD147 and then the subsequent rapid activation of the β_2_AR, which ultimately enhances and stabilizes bacterial adhesion.

## Mechanisms of TFP-Promoted βarr-Biased Activation of the β_2_AR

Initial investigations showed that the βarr-biased activation of the β_2_AR by PilE and PilV components of TFP was allosteric, since a pre-incubated orthosteric antagonist of the receptor did not block it, and likely involved the extracellular N-terminal region of the receptor (27). Homogeneous time resolved FRET experiments with purified PilE and PilV confirmed their specific direct interaction with this region of the receptor (30). Intriguingly, the amino-acid composition of β_2_AR N-terminus is conserved in mammals and almost identical for some species to that of human β_2_AR, contrasting with the strict human specificity of meningococcus. Moreover, in a fully reconstituted cellular model of meningococcus signalling, the mouse β_2_AR was activated by the pathogen in cells of human origin, whereas in a symmetrical experiment the human β_2_AR was not stimulated in mouse cells. These findings suggested that host factors, independent of the amino-acid composition, were involved in meningococcus interaction/activation of β_2_AR. The N-terminus of the β_2_AR also contains two asparagine-branched glycan chains, which were then investigated as potential alternative pilin binding sites. In the β_2_AR sequence the asparagine residues from which the glycan chains arise are separated by 9 amino-acid positions. When the two consensus N-glycosylation sites of the β_2_AR were introduced in the sequence of the angiotensin AT1R receptor, which is also expressed in endothelial cells but cannot be stimulated by meningococcus, the resulting chimera became activatable by the pathogen. Interestingly, not only the number but also the distance of the glycan chains in the N-terminus sequence was critical for receptor activation (30). A panel of lectins (i.e. proteins that exhibit high avidity for glycoprotein- and/or glycolipid-associated carbohydrates) were then pre-incubated with endothelial cells to block the specific glycan(s) involved in the interaction between the β_2_AR and meningococcus. *Nm* signalling was exclusively inhibited by lectins with the capacity of binding to terminal sialic acid (or N-acetyl neuraminic acid). Supporting a role of sialic acid in the interaction of N-terminal glycans with meningococcus, biochemical studies on purified N-terminal β_2_AR domain confirmed that both glycan chains contain sialic acid, whereas the inhibition of sialyl transferases (the enzymes involved in the addition of sialic acid to the glycan chain) prevented *Nm* signalling. These findings were particularly interesting in the context of meningococcus species selectivity. Two principal forms of sialic acids are found in mammals, N-Acetylneuraminic acid (Neu5Ac) and N-Glycolylneuraminic acid (Neu5Gc), which differ by a single oxygen atom (31). In mammals, Neu5Gc is predominant, whereas humans mainly express Neu5Ac (32). Neu5Gc is actually synthesised from Neu5Ac by the cytidine monophosphate-N-acetylneuraminic acid hydrolase (CMAH), which is absent in humans, due to a mutation in the *CMAH* gene (33). The hypothesis that the difference in sialic acids might contribute to the species selectivity was confirmed experimentally. Indeed, the deletion of *CMAH* in mouse endothelial cells restored meningococcal signalling *via* the β_2_AR (30). The nature of the human N-glycan recognized by *Nm* was further investigated using recombinant CD147 receptors, confirming the role of a complex sialilated N-glycan devoid of fucose residues (34).

Although it had been established that host-pathogen interactions can involve host cell glycans (35) and that the signalling properties of some receptors can be modulated by glycosylation (36, 37), the activation of a GPCR *via* the interaction between a ligand and N-glycan chains had not been reported previously. On the other hand, in addition to their usual role of chemosensors, some GPCRs can be activated by mechanical cues that are transduced by the receptor into intracellular chemical signals (reviewed in (38)), raising the hypothesis that mechanical forces applied *via* meningococcal TFP to β_2_ARs might similarly produce their activation (**Figure 1**). Indeed, bacteria growing at the cell surface of endothelial cells are permanently submitted to forces exerted by blood flow. In addition, TFP retraction, powered by the PilT ATPase can generate traction forces independently of hemodynamic flow (13, 39). Wild type and mutant meningococci deficient in PilT activity [Δ*pillT* mutants (40)] were compared for their capacity to induce signalling in hCMEC/D3 cells. Under basal conditions ezrin recruitment (as a marker of the cortical plaque formation) was significantly impaired under Δ*pillT* mutants compared to wild type *Nm*. The application of centrifugal forces on bacteria enhanced ezrin accumulation under both wild type and Δ*pillT* colonies, although the effect was significantly larger for wild type bacteria. Maximal signalling was thus obtained with the additive effect of PilT-induced pilus retraction and exogenous forces applied to meningcocci (30).

To confirm that pulling forces transmitted *via* N-glycans are sufficient to activate β_2_AR signalling agarose beads coated with lectins that specifically recognize Neu5Ac were used in a reconstituted cell system. After incubation on cell monolayers to allow lectin binding to sialic acid-terminated N-glycan chains of the β_2_AR the beads were submitted to orbital rotation. Whereas under basal conditions, only background accumulation of ezrin (used as activation readout) was observed under the beads, rotation appliance significantly enhanced ezrin accumulation but only when exogenous β_2_AR and βarrs were co-expressed in the cells (30).

## Discussion and Perspectives

**Figure 2:**
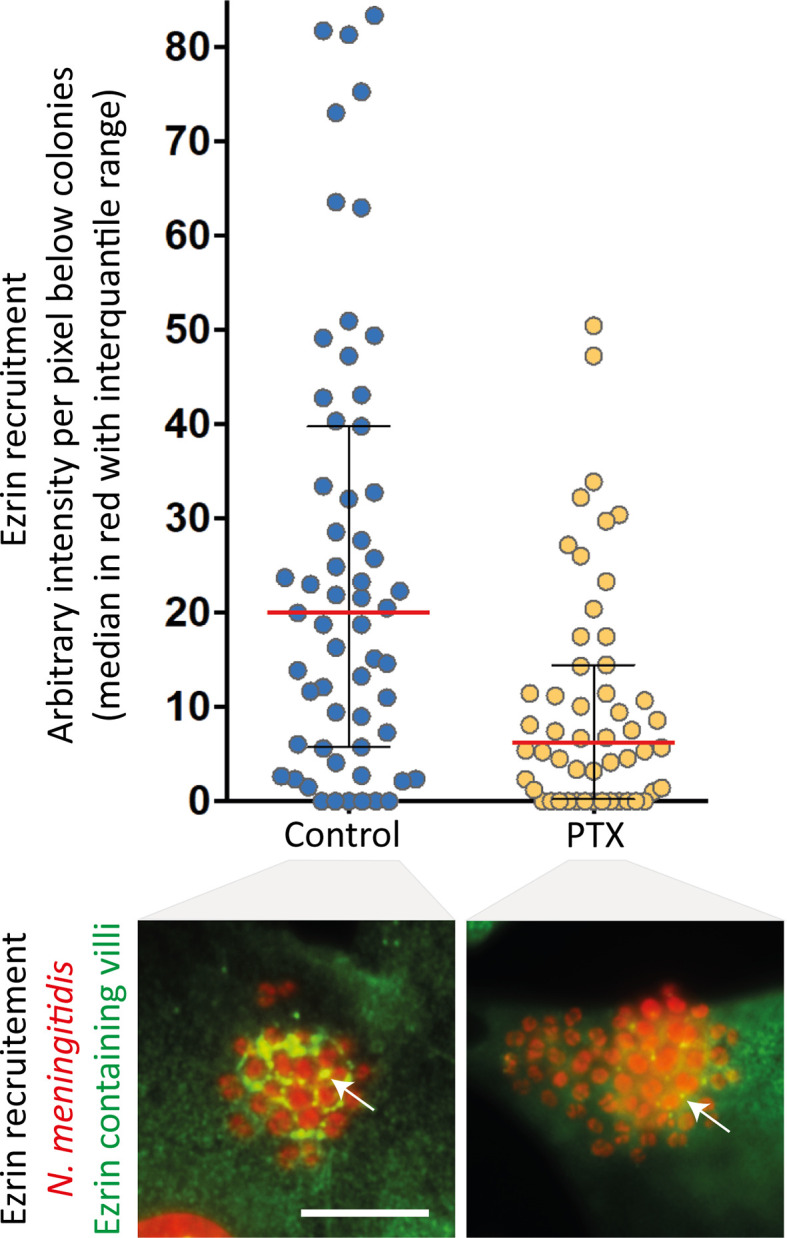
Pertussis toxin inhibits meningococcus-induced signalling. Endothelial hCMEC/D3 cells were infected with wild type *N. meningitidis* and submitted to centrifugal forces with or without pertussis toxin pre-incubation (PTX - 20ng/ml). Cells were then processed for the analysis of ezrin recruitment under bacterial colonies. Top panel: the intensity of ezrin recruitment was quantified for each individual colony and expressed as arbitrary units per pixel under colonies (as in 30). Each dot represents one colony. Median values (in red) with interquartile range are shown. A Mann-Whitney test was used to determine significant differences between median values. p=0.0002. Bottom panel: Representative immunofluorescence images. HCMEC/D3 cell nuclei and bacterial DNA (small, grouped dots) were labelled with DAPI (red); anti-ezrin antibodies stained ezrin (green). Arrows pointed at ezrin-enriched villi. Bar: 10µm.

In addition to their well-established role of chemosensors, which transduce catecholamine stimulation, β_2_AR can also function as mechanosensors for the traction forces applied on their N-terminal glycan chains. The signalling output produced by mechanical stimulation appears biased toward βarr-dependent pathways, since the receptor cognate Gs protein is not activated. However, in the presence of particular ligands, GPGRs can switch their coupling to a non-cognate G protein, likely because these ligands stabilize a unique receptor conformation that is competent for this unusual coupling. For example, carvedilol, an inverse-agonist of the classically Gs-coupled β_1_-adrenergic receptor (β_1_AR), was reported to promote its coupling to the inhibitory G protein Gi, in addition to the recruitment of βarrs (41). Moreover, this study demonstrated the existence of interplay between Gi and βarrs in the activation of the ERK1/2 MAP kinase pathway. Cell pre-incubation with pertussis toxin (PTX), which prevents Gi from interacting with GPCRs, partially inhibited meningococcus-induced signalling (**Figure 2**), suggesting that the mechanical traction forces on glycan chains similarly stabilize a unique β_2_AR active conformation capable of synergistic Gi coupling and activation of βarr signalling. This or a close conformation is likely also obtained by applying traction forces *via* beads that are coated with lectins specific for the exposed sialic acid of receptor glycan chains. Finally, the effect of PTX is consistent with previous studies indicating that signalling events attributed to βarrs might actually require some G protein contribution (42).

Although there is no evidence at the moment that, in addition to the pathophysiological role in meningococcal infection, this mode of activation of the β_2_AR is involved in physiological processes, this hypothesis merits further investigations. Indeed, many cell types express integral lectins as structural components of their plasma membrane. Among them, I-type lectins are glycan-binding proteins in which the binding domain is homologous to immunoglobulin superfamily proteins. The Siglec family is a subgroup of I-type lectins, which all recognizes terminal sialic acids with specificity for adjacent carbohydrates within the glycan chain. Most Siglecs have one or more tyrosine-based signalling motifs in their cytoplasmic tails, or associate with membrane adaptor proteins containing cytosolic tyrosine motifs. Following their tyrosine phosphorylation by Src family kinases, they recruit and activate SH2-domain-containing effectors (43). Siglecs are expressed by blood cells, microglia, osteoclasts, myelin forming cells, and placental trophoblasts. Macrophages, adhere to and roll over endothelial cells before stopping under blood flow and penetrate into tissues. They express at the cell surface Siglec-1 (35), which displays the same glycan specificity as Mal-I, one of the lectins that block meningococcal signalling. In this context Siglec-1 might bind to the β_2_AR and induce signalling pathways contributing to macrophage diapedesis. Also, signalling promoted by cell-to-cell interactions are essential during development, during which sialic-acids are known to play a major role, as shown by early embryonic lethality in case of disruption of sialic acid synthesis (43).

More generally, GPCRs are almost ubiquitous and 80% of them display one or more N-glycan chains, which might similarly be involved in the interaction with surrounding cells through lectins. Although, it is difficult at the moment to evaluate how many GPCRs could actually be activated *via* a traction applied on their N-glycans, β_2_AR might not be a unique example, since the angiotensin AT1R engineered to express a second glycan chain in its N-terminus can be activated by meningococcus.

## Data Availability Statement

The raw data supporting the conclusions of this article will be made available by the authors, without undue reservation.

## Author Contributions

SM wrote the first version of the manuscript, which was reviewed and corrected by the co-authors. All authors contributed to the article and approved the submitted version.

## Funding

This article was supported by ANR-14-IFEC-0006-01, ANR-15-CE15-0002 and ANR-19-CE14-0045 grants. SM and MC are also supported by the INSERM, the CNRS, and the Université de Paris.

## Conflict of Interest

The authors declare that the research was conducted in the absence of any commercial or financial relationships that could be construed as a potential conflict of interest.

## Publisher’s Note

All claims expressed in this article are solely those of the authors and do not necessarily represent those of their affiliated organizations, or those of the publisher, the editors and the reviewers. Any product that may be evaluated in this article, or claim that may be made by its manufacturer, is not guaranteed or endorsed by the publisher.
